# Aortic endograft infections have worse outcomes compared to aortic surgical grafts or primary mycotic aortic infections

**DOI:** 10.1016/j.jvs.2025.06.011

**Published:** 2025-06-18

**Authors:** Bowen Xie, Dana B. Semaan, Nicole Alindogan, Georges E. Al-Khoury, Michael J. Singh, Edith Tzeng, Michel S. Makaroun, Mohammad H. Eslami, Rabih A. Chaer

**Affiliations:** a Division of Vascular/Endovascular Surgery, UT Health San Antonio, San Antonio; b Division of Vascular Surgery, University of Pittsburgh Medical Center, Pittsburgh; c Department of General Surgery, Allegheny Health Network, Pittsburgh; d Division of Vascular Surgery, Charleston Area Medical Center Vascular Center of Excellence, Charleston; e Division of Vascular & Endovascular Surgery, Stony Brook Medicine, Stony Brook.

**Keywords:** Infected aortic grafts, Outcomes, Primary aortic infections

## Abstract

**Background:**

Aortic infection is associated with high morbidity and mortality, but the outcomes of prosthetic (surgical or endovascular) grafts compared with primary aortic infections are poorly defined. This large single-center retrospective study aims to compare the outcomes of primary and prosthetic aortic graft infections.

**Methods:**

Patients diagnosed with primary or infected aortic grafts between January 2000 and December 2022 were included. Patients were grouped based on the type of infection (primary, surgical graft, and endograft). Baseline demographics, symptoms, imaging, interventions, and outcomes were evaluated. Primary outcomes include overall and reintervention-free survival. Kaplan-Meier survival and Cox proportional hazards analysis were performed.

**Results:**

A total of 145 patients presented during the study period with primary infection of the native aorta (33.8%) or aortic graft infection (66.2%: 44.8% surgical graft and 21.4% endograft). In-hospital mortality (39% vs 15% vs 10%; *P* = .007) and 30-day complication rates (68% vs 40% vs 37%; *P* = .015) were highest among endografts, followed by surgical grafts, and lowest among primary infections, respectively. Primary aortic infections had the highest 30-day, 1-year, and 5-year survival on Kaplan-Meier analysis compared with surgical and endograft infections. Reintervention-free survival was also highest in primary infections at 1 and 5 years (log-rank *P* < .001). On multivariate analysis, infected surgical grafts and endografts were associated with a significantly higher 30-day mortality (hazard ratio, 8.1; *P* = .016 and hazard ratio, 5.8; *P* = .035, respectively). Visceral artery involvement was a major determinant of mortality at 1 year and 5 years but not at 30 days, whereas treatment type did not affect mortality across all groups. There was no difference in the duration of intravenous antibiotic treatment between groups (42 vs 45 vs 44 days; *P* = .68).

**Conclusions:**

Infected aortic endografts have lower short- and long-term survival as well as a lower rate of long-term intervention-free survival when compared with surgical graft and primary aortic infections. Visceral artery involvement was associated with increased mortality. Predictors of endograft infection need to be determined to minimize complications, and careful consideration is necessary before endovascular aortic interventions if there is concern an underlying infectious process.

Although rare, aortic infections are often associated with poor prognosis. The increased rate of expansion as well as the risk of rupture often result in high morbidity and mortality despite intervention.^1,2^ Surgical treatment can consist of open repair using either autogenous or prosthetic/cadaveric conduit, extra-anatomic bypass and staged aortic ligation, or endovascular exclusion of the segment of infected aorta.

As the prevalence of open surgical and endovascular repair of aortic pathology increases so too does the risk of prosthetic aortic graft infections.^3^ These present a difficult and potentially more challenging problem to address when compared with native aortic infections as complete removal of the infected graft may prove to be more difficult in the setting of extensive aortic involvement and potentially worse medical comorbidities. Although there has been a plethora of data regarding the treatment and outcomes of native aortic infections,^1,4,5^ less is known about the outcomes of infected prosthetic grafts (surgical or endovascular) in comparison to primary infections.

This large single-institution retrospective study serves to compare the outcomes of native vs prosthetic (surgical or endovascular) aortic graft infections and identify predictors affecting mortality.

## METHODS

### Patient cohort and data source.

This is a retrospective cohort study of all patients diagnosed with an aortic infection involving the abdominal or thoracoabdominal aorta between January of 2000 and December of 2022. Patients with an isolated thoracic aortic infection were excluded. This study was approved by the University of Pittsburgh Institutional Review Board (STUDY22100124). We included all adult patients (≥18 years) who were diagnosed with either a primary aortic infection or with an infected aortic graft by searching the electronic health records (EHRs). Patients with asymptomatic aortitis or inflammatory aneurysms were not included in the search. The study followed Strengthening the Reporting of Observational studies in Epidemiology (STROBE) guidelines.^6^

Individual patients, rather than infectious episodes, were identified and categorized based on the index operation. There was no crossover between treatment groups. Primary aortic infection was defined as evidence of an aortic aneurysm/pseudoaneurysm either radiologically (computed tomography angiography [CTA] scans) or surgically (evidence of aneurysm intraoperatively) in addition to evidence of infection with one of the following criteria: (1) clinical presentation (fever ≥38C or concomitant or recent infection); (2) laboratory tests (elevation of white blood cell count or C-reactive protein, or positive cultures); (3) radiological findings on CTA (rapid expansion of aneurysm, multi-lobular aneurysms/eccentric aneurysms, periaortic gas, and periaortic soft tissue mass/stranding); (4) surgical evidence (purulence and inflammation found intraoperatively). Additionally, patients in the primary aortic infection group were defined as having no previous aortic repair. Patients with infected aortic grafts were defined similarly as the primary aortic infections group with the addition of a history of aorticrepair with either a surgical graft or endograft. Patients without an aneurysmal degeneration but with obvious signs of infection on CT, such as the presence of a peri-aortic inflammatory phlegmon, or gas, were also included. Some patients who underwent the index operation at another institution were transferred to our center upon diagnosis for further care and were included if detailed information about their prior care was available. Additionally, those patients did not undergo intervention prior to transfer except for initiation of antibiotic therapy upon transfer. Patients with nonaortic infections (groin infections, endocarditis, arteriovenous graft infections, and lower extremity bypass graft infections), noninfected aortic aneurysms, and those with missing information in the charts were excluded from the cohort.

Patient data was extracted from the EHR by chart review and included baseline demographics, clinical features, anatomic features, type of management, culture results, and short- and long-term outcomes.

Baseline demographics included age, sex, race, comorbidities, recent nonaortic surgery within 2 months, recent infection within 3 weeks, and recent or ongoing antibiotic use within 3 weeks to determine potential sources for aortic infection. Two months were selected instead of 30 days to make sure to capture any subacute perioperative infection related to any recent nonaortic surgery. Clinical features included presenting symptoms (asymptomatic, abdominal pain, back pain, hematemesis, melena/gastrointestinal bleed, groin symptoms [swelling, pain, or bleeding], fever, constitutional symptoms [night sweats, fatigue, weight loss, or decreased appetite], lower limb symptoms [claudication, pain, weakness, or paresthesia]), hemodynamics on presentation (heart rate, systolic blood pressure, and diastolic blood pressure), temperature at presentation, and laboratory results (white blood cell count, hemoglobin, C-reactive protein, and creatinine).

Anatomic features included the involvement of the visceral arteries, presence of a pseudoaneurysm, presence of rupture or contained rupture, presence and size of a collection or abscess, and aneurysm size, shape (saccular or fusiform), and location (suprarenal, pararenal, or infrarenal). These features were based on CTA scans.

Management included surgical repair (open in situ aneurysm repair [OAR], extra-anatomic repair [EAR], or endovascular aortic repair [EVAR]), medical treatment with antibiotics, palliative care, or other (washout and debridement, repair of aorto-enteric fistula with installation of intraabdominal antibiotic bioabsorbable beads, and thoracic endovascular aortic repair with 4-vessel debranching of the visceral arteries). There are no institution-wide recommendations outlining the treatment of aortic infection, nor recommendations regarding the use of palliative EVAR, and the decision was at the discretion of the attending surgeon. All patients had a senior surgeon involved with their operative intervention either as a primary or assistant surgeon. EAR consisted of an axillary to femoral bypass with subsequent explantation of the infected graft or resection of the infected aneurysm and ligation of the remaining aorta both proximally and distally. EVAR consisted of relining the area of defect/infection with a bifurcated or tube endograft, typically as a bridge to open repair but sometimes as a definitive repair in patients at high risk for open repair. Patient with infected surgical grafts or endografts were treated with graft explanation with supra-celiac clamping, followed by OAR or EAR. The type of grafts used for OAR included rifampin-soaked grafts, cryopreserved aorta, or autologous femoral vein. Information on the degree of contamination was also collected for patients undergoing OAR or EAR by findings of severe inflammation, purulence or aorto-enteric fistula from the operative reports. Cultures included those taken from blood, infected aortic tissue, or other sites (wound, sputum, cerebrospinal fluid, pleural fluid, groin wound, peritoneal fluid, urine, and lumbar discs), and their results were classified as Gram-stain positive, Gram-stain negative, fungal, or no growth. Intravenous antibiotic therapy was used for at least 48 hours prior to aortic reintervention in stable patients, and for at least 6 weeks postoperatively.

Date of death and cause of death were identified using the Social Security Death index, last stated update on December 6, 2022, which is linked to the EHR. Cause of death was classified as related to aortic infection (hemorrhagic shock, septic shock, or mesenteric ischemia), unrelated to aortic infection (respiratory failure [pneumonia], neurologic [intracranial hemorrhage, stroke, and encephalopathy], septic shock [due to unknown source or endocarditis], hemorrhagic shock [due to retroperitoneal bleed or gastrointestinal bleed], metastatic cancer, mesenteric ischemia, and myocardial infarction), or unknown.

### Outcomes.

Short-term outcomes of interest included 30-day major complications, in-hospital mortality, 30-day readmission, 30-day all-cause mortality, and 30-day disease-related mortality. Major complications included respiratory (atelectasis, pneumonia, or respiratory failure requiring intubation), renal (acute kidney injury or renal failure requiring dialysis), gastrointestinal (bleeding, ischemia, or liver failure), cardiac (myocardial infarction, congestive heart failure, or cardiac arrest), vascular (surgical site bleeding, intra-abdominal bleeding, limb ischemia, or venous thromboembolisms [deep vein thrombosis or pulmonary embolism]), and infectious (intra-abdominal abscess or extra-abdominal infection/abscess). Disease-related mortality included mortality due to hemorrhagic shock, septic shock, or mesenteric ischemia as defined above.

Long-term outcomes included overall survival, disease-related mortality, and reintervention-free survival at 1 and 5 years. We defined reintervention as any procedure performed to address complications secondary to the index management, including recurrent graft infections, bleeding, endoleaks, groin infections, limb ischemia, and pseudoaneurysm.

### Statistical analysis.

Baseline demographics were presented as mean (± standard deviation) when normally distributed and median (interquartile range [IQR]) when skewed for continuous data and compared using Student’s *t*-test. Categorical data were presented as number (%) and compared using Fisher’s exact test. Kaplan-Meier survival curves were created for mortality and reintervention outcomes at 30 days, 1 year, and 5 years and compared using log-rank testing. Multivariable Cox regression analyses were performed to assess the determinants of disease-related mortality at 30 days, 1 year, and 5 years. Cox regression analysis produced adjusted hazard ratios (aHRs) with 95% confidence intervals (CIs). A *P* value ≤ .05 indicates a statistical significance. Statistical analysis was performed using Stata SE 17.0 (StataCorp).

### Subgroup analysis.

To investigate the effect of treatment type on outcomes of patients with infected grafts, a subgroup analysis was performed on this subgroup of patients. The subgroup was grouped by treatment type (OAR, EAR, EVAR, and other). Assessed outcomes included all-cause mortality and disease-related mortality at 30 days, 1 year, and 5 years. Reintervention-free survival was also assessed at 1 year and 5 years. Kaplan-Meier survival analysis and multivariate Cox regression were performed.

## RESULTS

### Crude comparison.

The EHR search yielded an index cohort of 452 patients, of whom 145 met the inclusion criteria (Supplementary Fig 1, online only). Primary infections/mycotic aortic aneurysms (MAAs) made up 33.8% of the cohort, whereas infected endografts made up 21.4%, and the majority of the cohort presented with an infected surgical graft (44.8%). Patients in the endograft group were significantly older and less likely to have a history of recent infection within 3 weeks of presentation, but more likely to present with recent antibiotic use. The endograft group more often presented with fevers and a fusiform aneurysm. Ruptures were less commonly seen in both the surgical and the endograft groups (6% and 14%, respectively) but were the most prevalent among primary infection/MAAs (52%) (Table I). There was a significant difference in the type of treatment received between the groups. OAR was most commonly performed among patients with a primary infection/MAA (34.7%), whereas those with an infected endograft were more likely to receive an EAR (51.6%; *P* = .002). The most common type of bacteria isolated from surgical samples were Gram-positive organisms for the primary infections and endograft groups (Supplementary Table I, online only). Gram-positive organisms were also the most commonly isolated blood culture organisms across all groups. There was no difference in the total duration of antibiotic treatment between the groups (42 vs 45 vs 44 days; *P* = .68), the majority of which were intravenous throughout its entirety.

### Short-term outcomes.

**Fig 1.**
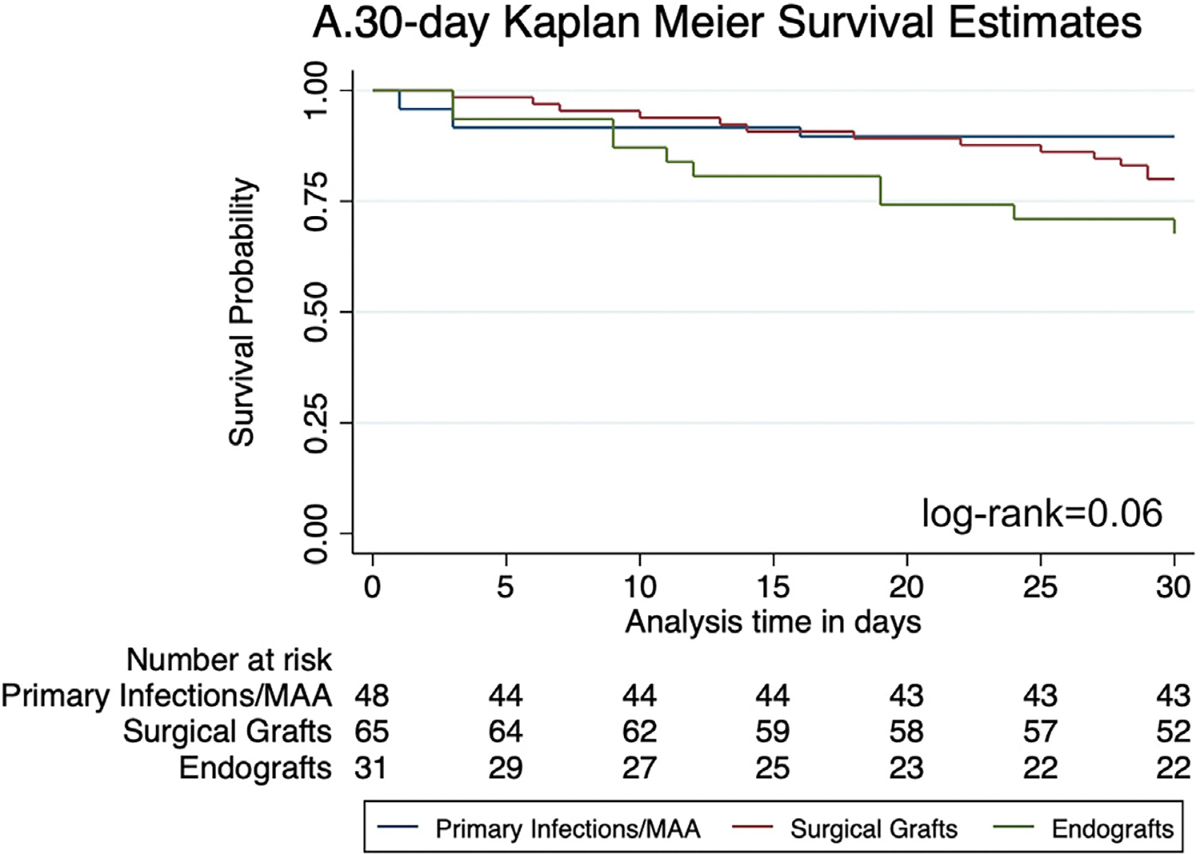
Thirty-day overall survival.

The rate of major complications at 30 days was significantly different between the three groups, with the endograft group having the highest rate (68%) and primary infection/MAA group having the lowest (37%; *P* = .015). The same was seen for in-hospital mortality (Table II), which more commonly occurred in the endograft group (39%) and least commonly in the primary infections/MAA group (10%; *P* = .007). There was no difference in the 30-day readmission rates; however, patients in the endograft group more often suffered from disease-related death (70%; *P* = .027). The most common form of disease-related death consisted of multi-organ systems failure secondary to septic shock from aortic infection (79%). There was no significant difference among the groups for 30-day overall survival (*P* = .06) (Fig 1). Using the primary infection/MAA group as a reference, multivariate Cox regression showed a higher risk for 30-day mortality among both the surgical graft and endograft group (aHR, 8.1; 95% CI, 1.47–44.03; *P* = .016 and aHR, 5.8; 95% CI, 1.13–29.32; *P* = .035, respectively), whereas no other significant determinants of 30-day mortality were seen (Supplementary Table II, online only). As for 30-day disease-related mortality, the presence of an infected endograft (aHR, 5.7; 95% CI, 1.09–29.18; *P* = .038) and visceral involvement (aHR, 4.3; 95% CI, 1.05–17.61; *P* = .042) were the only significant determinants observed.

### Long-term outcomes.

**Fig 2.**
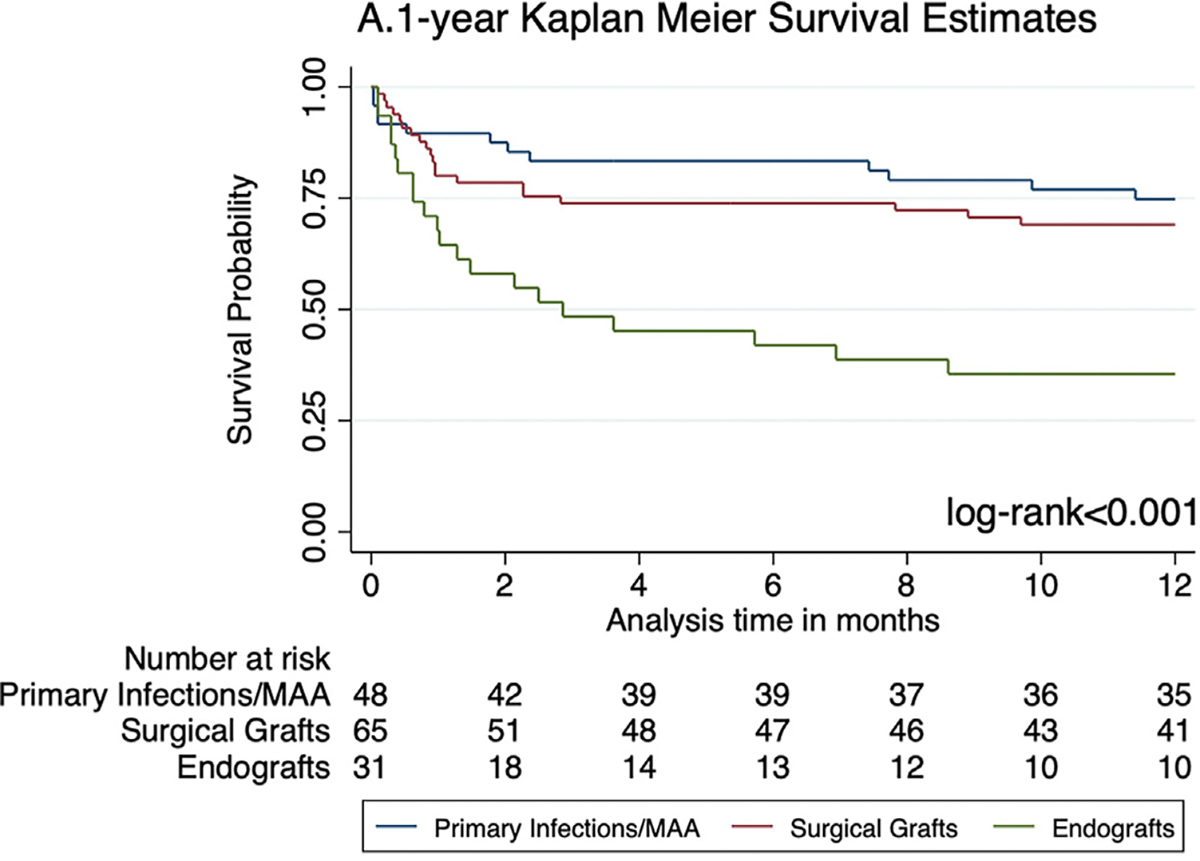
One-year overall survival.

**Fig 3.**
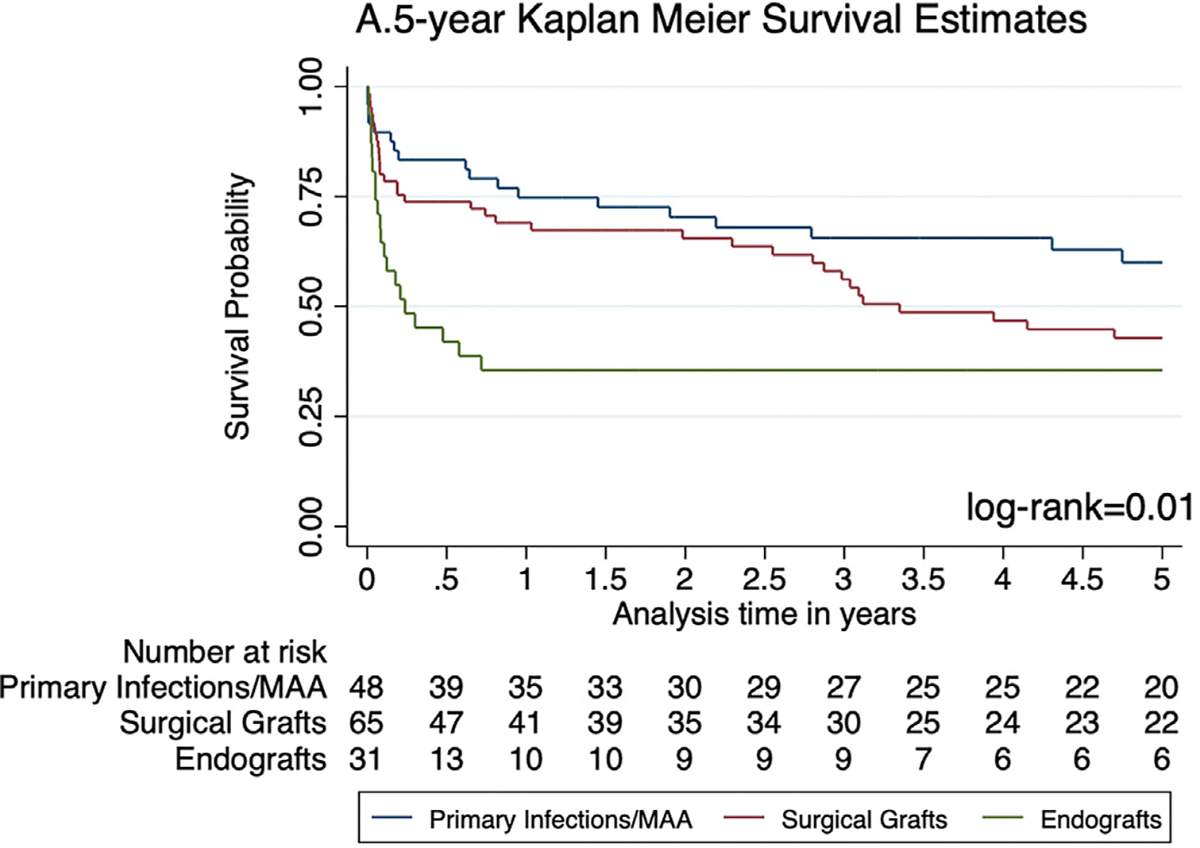
Five-year overall survival.

Patients with infected endografts had significantly lower 1-year overall survival (35.5% ± 0.09%) than both surgical graft infections (69.1% ± 0.06%) and primary infections/MAA (74.8% ± 0.06%) (Fig 2). Similar results were observed for 5-year overall survival (endograft group: 35.5% ± 0.09%; surgical graft group: 42.8% 6 0.07%; primary infections/MAA: 60.1% ± 0.07%), with the largest difference manifesting within the first year (Fig 3). Reintervention-free survival was lowest among the endograft group at both 1 year (20% ± 0.07%; log-rank < .001) and 5 years (16% ± 0.07%; log-rank < .001) (Supplementary Fig 2, online only).

### Adjusted analysis.

When assessing the factors associated with 1-year all-cause mortality after controlling for visceral artery involvement, treatment type, and surgical and blood culture results, both infected surgical grafts (aHR, 3.7; 95% CI, 1.07–12.51; *P* = .039) and infected endografts (aHR, 4.1; 95% CI, 1.31–12.61; *P* = .016) had an increased risk of mortality compared with primary infections/MAAs. Involvement of the visceral arteries also significantly increased the mortality risk (aHR, 3.9; 95% CI, 1.35–11.41; *P* = .012) (Table III). Having a Gram-positive organism on surgical culture was also a significant determinant of 1-year disease-related mortality (aHR, 4.4; 95% CI, 1.05–18.61; *P* = .042), although there was still a large cohort of patients with no positive surgical or blood cultures across all treatment groups (Supplementary Table I, online only). Similar results were seen for 5-year all-cause mortality, with the surgical graft group and endograft group having a higher mortality risk than primary infections/MAAs (aHR, 7.7; 95% CI, 2.57–22.81; *P* < .001 and aHR, 4.7; 95% CI, 1.66–12.99; *P* = .003, respectively). In addition to visceral involvement, treatment with EVAR was also associated with increased mortality (aHR, 8.1; 95% CI, 1.41–46.25; *P* = .019), as was the growth of Gram-positive and Gram-negative organisms on blood culture (Table III).

### Subgroup analysis.

For patients with infected surgical and endografts, the most common treatment was EAR (48.9%) followed by OAR, other, and EVAR (21.9%, 19.8%, and 9.4%, respectively). Those who received an EVAR were more likely to have presented with a rupture (38%; *P* = .04) when compared with the other treatment modalities (Supplementary Table III, online only). There was no difference in overall survival between groups, and the only determinant of mortality was involvement of the visceral arteries.

## DISCUSSION

Our series represents a large single-institution experience comparing the outcomes after treatment of varying types of aortic infections (native vs surgical graft vs endograft). Explantation of aortic stent grafts is often associated with higher morbidity and mortality due to the need for suprarenal or supra-celiac clamp, the presence of suprarenal fixation or other proximal fixation adjuncts, and management of the iliac limbs, in addition to already pre-existing comorbidities present within the patient population.^7,8^

We demonstrated an increased mortality associated with treatment of infected aortic endografts as compared with both primary infection/mycotic aneurysms and infected surgical grafts both in the short-term and long-term post-operative period (Table II and Table III). The rate of in-hospital mortality for infected endografts of 39% in our cohort was similar to other in-hospital and early mortality documented in literature.^3,9–12^ Multisystem organ failure secondary to septic shock from surgical intervention was the most common cause of disease-related death at 79%. The mortality difference seen may be due to indolence associated with graft infections, with a significantly lower proportion of surgical and endografts presenting without any evidence of recent infection (Table I), thus allowing longer microbial incubation time and larger extent of invasion within the aorta. In addition, although the graft explant surgical technique was similar between the two groups in terms of supra-celiac clamping, the aortic neck is more challenging with infected endografts, especially in patients with suprarenal fixation, with typically no infrarenal neck and/or a thinned-out aortic wall compared with surgical grafts where the aortic anastomosis is sometimes quite distal to the renal arteries. Additionally, patients who underwent an EVAR as their index operation could have been higher risk at baseline to be offered a minimally invasive repair, which could explain their worse outcomes. When comparing presentation status and operative complexity, there was no significant difference in vital signs or operating time and estimated blood loss (EBL) between infection groups.

When looking at mid-term and long-term survival, 1-year and 5-year overall survival were also significantly lower in the aortic endograft group compared with both surgical grafts and native aortas. These differences appear to manifest most importantly within the first year as demonstrated by our Kaplan-Meier analysis (Fig 3). This serves to highlight the devastating nature of aortic graft infections, with the index insult from surgical intervention as the most impactful on long-term survival. Multivariate analysis of mortality across all time points (30 days, 1 year, and 5 years) demonstrates the presence of an endograft as one of the only significant risk factors along with involvement of the visceral arteries, once again highlighting the poor outcomes associated with aortic stent graft infection.

Our results highlight the variety of management techniques for aortic infections, ranging from in-situ reconstruction and extra-atomic bypass to endovascular stenting and medical management. Those who presented with a native aortic infection or mycotic aneurysm were most commonly treated by OAR (35%), followed by EVAR (33%), whereas those who presented with surgical or endograft infections were primarily treated with EAR via axillo-bifemoral bypass and graft explant (48% and 52%, respectively), with the majority of patients presenting with an infrarenal pathology and no significant difference in the anatomic location (Table I). Medical management with long-term antibiotic therapy was only undertaken in the case of surgical or endograft infection (Supplementary Table I, online only). Although Berard et al have shown that selective use of in situ reconstruction provides an adequate treatment modality for native aortic infections,^4^ Sörelius et al have demonstrated a shift in the treatment of mycotic aneurysms towards EVAR in recent years with no difference in long-term survival.^1,2^ Other studies have also shown EVAR to be comparable to open surgical reconstruction in early outcomes.^5,13^ This trend is similarly reflected in our patient population with a near equal proportion of primary aortic infections undergoing OAR vs EVAR. Similar rates of perioperative morbidity and mortality have been demonstrated when comparing extra-anatomic bypass with in-situ reconstruction for the treatment of aortic graft infections.^3,12,14–17^ In our series, extra-anatomic bypass was more common compared with in-line reconstruction in the setting of both surgical and endograft infections (48% and 52% vs 20% and 26%, respectively); however, there was no difference in short-term or long-term survival based on the type of reconstruction performed (Supplementary Tables II and III, online only).

Although Gram-positive cultures appear to be the most common organisms isolated from both surgical and blood cultures in all forms of aortic infections, there were still a significant portion of patients who had no growth on any laboratory cultures in all three groups (Supplementary Table I, online only). Interestingly, although Smed et al demonstrated decreased survival associated with Gram-negative infections as well as a higher proportion of polymicrobial surgical cultures,^3^ our cohort demonstrated no incidence of polymicrobial surgical cultures within the primary aortic infection group and only as a minority in both the surgical and endograft groups. Although we demonstrated no difference in 30-day disease-related mortality on multivariate analysis with organisms identified on either blood or surgical cultures, there does appear to be a mortality signal associated with Gram-negative blood cultures in long-term disease-related mortality (Table III). Despite these findings, there still appears to be no difference in the duration of antibiotic treatment between native vs surgical vs endograft infections.

Our series highlights the devastating nature of aortic infections, particularly with endograft infections as compared with both native aortic and surgical graft infections. The increased mortality signal appears most prominent within 30 days to 1 year, likely arising from the highly morbid complications secondary to surgical intervention despite similarities in the extent of infection on presentation and surgical technique. Native aortic infections appear more prone to rupture on presentation with a higher rate of endo-salvage or in-line reconstruction, whereas the graft infections (both surgical and endografts) are most often treated with extra-anatomic bypasses. Despite the differences in treatment modalities, there still appears to be no difference in short- or long-term survival across the different treatment groups. In addition, despite the stark increase in mortality associated with endograft infections, there still does not appear to be a difference in the duration of antibiotic regimens between groups. This may be partially attributed to the lack of definitive causative organisms on surgical or blood cultures, highlighting the need for a potentially more aggressive approach to microbial sterilization in the setting of endograft infection.

There are several limitations to our study. First, given the retrospective nature, index antibiotic therapy at transferring facilities may not be completely captured and may lead to sterilization of cultures prior to definitive treatment, thus skewing the association between the causative organism and outcomes. In addition, the rarity of aortic infection also prevents adequate matching across treatment arms or the categorization of the types of endografts explanted to appropriately delineate the difference in mortality seen with aortic stent graft infections. Furthermore, the lack of standardization to quantify the extent of intraoperative infection could create further bias in determining outcomes among the various types of aortic infection. Finally, comparison with a control noninfectious aortic graft group was not made, and thus morbidity and mortality risks associated with infected aortic grafts (surgical or endografts) may be artificially inflated.

## CONCLUSIONS

When comparing aortic infections (primary vs surgical vs endografts), endograft infections appear to be the most devastating with the lowest rate of short-term and long-term survival, whereas primary aortic infections appear to be the most conducive to survival. On multivariate analysis, visceral artery involvement and endograft infection were persistently associated with increased long-term mortality. A more extensive analysis of the predictors of endograft infection is needed to minimize the potential long-term complications of endovascular aortic interventions, and judicious attention to sterile technique should be employed as the outcomes of endograft infections appear to be devastating.

## Supplementary Material

Supp Figure 1

Supp Figure 2

Supp Table I

Supp Table II

Supp Table III

Additional material for this article may be found online at www.jvascsurg.org.

## Figures and Tables

**Table I.** Baseline demographics by type of infection

	Primary infections/MAAs n = 49 (33.8%)	Surgical grafts n = 65 (44.8%)	Endografts n = 31 (21.4%)	*P* value
Age, years	64.8 (9.2)	67.8 (10.9)	72.9 (10.4)	.004
Gender				
Male	33 (67)	42 (65)	27 (87)	.059
Female	16 (33)	23 (35)	4 (13)	
Race				
Non-Hispanic White	43 (88)	61 (94)	25 (81)	.061
Black	5 (10)	1 (2)	2 (6)	
Other	1 (2)	3 (5)	4 (13)	
Peripheral artery disease	2 (4)	31 (48)	9 (29)	<.001
Hypertension	35 (71)	48 (74)	20 (65)	.61
Diabetes	15 (31)	9 (14)	3 (10)	.035
Chronic kidney disease	4 (8)	6 (9)	6 (19)	.26
Hyperlipidemia	21 (43)	26 (40)	15 (48)	.73
Coronary artery disease	15 (31)	19 (29)	16 (52)	.087
Congestive heart failure	7 (14)	7 (11)	0 (0)	.071
COPD	8 (16)	19 (29)	6 (19)	.24
Recent infection (within 3 weeks)	24 (49)	15 (23)	6 (19)	.004
Recent surgery (within 3 weeks)	1 (2)	8 (12)	3 (10)	.12
Antibiotics on presentation	1 (2)	17 (26)	10 (32)	<.001
Asymptomatic presentation	5 (10)	4 (6)	3 (10)	.68
Abdominal pain	29 (59)	21 (32)	12 (39)	.015
Back pain	26 (53)	6 (9)	10 (32)	<.001
Groin symptoms	0 (0)	15 (23)	2 (6)	<.001
Constitutional symptoms	9 (18)	13 (20)	10 (32)	.30
Fever	2 (4)	8 (12)	10 (32)	.002
Heart rate on presentation, bpm	90 (72–102)	86 (75–95)	84 (74–92)	.26
SBP on presentation, mmHg	128 (114–147)	130 (110–153)	128 (113–137)	.95
Temperature on presentation	36.7 (36.45–37.1)	36.7 (36.4–37)	36.8 (36.5–37.3)	.45
Rupture on CT	25 (52)	3 (6)	4 (14)	<.001
Pseudoaneurysm on CT	14 (29)	9 (17)	5 (19)	.38
Aneurysm size, mm	44 (32–52)	43.5 (34–61)	55 (40–65)	.18
Aneurysm shape				
Saccular	27 (69)	5 (45)	3 (27)	.030
Fusiform	12 (31)	6 (55)	8 (73)	
Aneurysm location				
Suprarenal	8 (16)	4 (6)	3 (10)	.15
Infrarenal	33 (67)	45 (70)	22 (71)	
Pararenal	7 (14)	6 (9)	5 (16)	
Thoracic	1 (2)	9 (14)	1 (3)	

*CT*, Computed tomography; *COPD*, chronic obstructive pulmonary disease; *MAA*, mycotic aortic aneurysm; *SBP*, systolic blood pressure.

Data are presented as number (%), mean (standard deviation), or median (interquartile range).

**Table II.** Complications and causes of death

	Primary infections/MAAs n = 49 (33.8%)	Surgical grafts n = 65 (44.8%)	Endografts n = 31 (21.4%)	*P* value
30-day major complication	18 (37)	26 (40)	21 (68)	.015
In-hospital mortality	5 (10)	10 (15)	12 (39)	.007
30-day readmission	12 (27)	11 (19)	3 (11)	.24
Cause of death				
Related to aneurysm	6 (26)	13 (32)	14 (70)	.027
Unrelated to sneurysm	5 (22)	11 (27)	3 (15)	
Unknown	12 (52)	17 (41)	3 (15)	

*MAA,* Mycotic aortic aneurysm.

Data are presented as number (%).

**Table III.** Multivariate Cox regression for all-cause mortality and disease-related mortality at 1 year and 5 years

	1-year all-cause mortality			1-year disease-related mortality		
	aHR	95%CI	*P* value	aHR	95%CI	*P* value

Primary infections/MAAs	Ref.	Ref.	Ref.	Ref.	Ref.	Ref.	
Surgical grafts	3.7	1.07–12.51	.039	4.9	0.88–27.87	.07
Endografts	4.1	1.31–12.61	.016	6.8	1.36–34.29	.02
Visceral involvement	3.9	1.35–11.41	.012	6.7	1.82–24.82	.004
Treatment						
OAR	Ref.	Ref.	Ref.	Ref.	Ref.	Ref.
EAR	1.8	0.72–4.33	.215	1.2	0.41–3.82	.695
EVAR	2.5	0.26–23.68	.424	2.3	0.23–24.05	.475
Other	0.3	0.03–2.79	.288	0.5	0.04–5.18	.542
Surgical culture results						
No growth	Ref.	Ref.	Ref.	Ref.	Ref.	Ref.
Gram-negative	1.1	0.31–3.85	.899	0.7	0.11–4.13	.652
Gram-positive	2.9	0.99–8.96	.051	4.4	1.06–18.61	.042
Mixed	1.9	0.64–6.18	.234	1.2	0.24–6.07	.822
Blood culture results						
No growth	Ref.	Ref.	Ref.	Ref.	Ref.	Ref.
Gram-negative	2.9	0.75–11.02	.124	5.8	0.99–33.87	.051
Gram-positive	2.1	0.58–6.88	.27	1.5	0.31–6.84	.63
	5-year all-cause mortality			5-year disease-related mortality		
	aHR	95%CI	*P* value	aHR	95%CI	*P* value

Primary infections/MAAs	Ref.	Ref.	Ref.	Ref.	Ref.	Ref.
Surgical grafts	7.7	2.57–22.81	0	3.9	0.81–19.7	.091
Endografts	4.7	1.66–12.99	.003	5.1	1.23–21.16	.024
Visceral involvement	3.9	1.39–11.17	.01	7.1	1.9–26.4	.003
Treatment						
OAR	Ref.	Ref.	Ref.	Ref.	Ref.	Ref.
EAR	1.7	0.79–3.67	.173	1.3	0.43–4.07	.625
EVAR	8.1	1.41–46.25	.019	4.99	0.78–32.11	.09
Other	0.3	0.05–1.54	.146	0.5	0.04–5.04	.522
Surgical culture results						
No growth	Ref.	Ref.	Ref.	Ref.	Ref.	Ref.
Gram-negative	0.6	0.22–1.87	.419	0.8	0.13–4.59	.77
Gram-positive	1.8	0.71–4.57	.212	4.3	0.99–18.21	.051
Mixed	1.33	0.51–3.53	.564	1.33	0.26–6.74	.729
Blood culture results						
No growth	Ref.	Ref.	Ref.	Ref.	Ref.	Ref.
Gram-negative	4.1	1.13–14.69	.031	7.1	1.28–38.34	.025
Gram-positive	3.98	1.25–12.7	.02	1.7	0.36–8.01	.509

*aHR*, Adjusted hazard ratio; *CI*, confidence interval; *EAR*, extra-anatomic repair; *EVAR*, endovascular aneurysm repair; *MAA*, mycotic aortic aneurysm; *OAR*, open aneurysm repair in situ; *Ref*, reference group.

## ARTICLE HIGHLIGHTS

- **Type of Research:** Retrospective, single-center, multi-hospital network cohort study
- **Key Findings:** Primary aortic infections had the highest 30-day, 1-year, and 5-year survival on Kaplan-Meier analysis compared with surgical and endograft infections. Infected surgical grafts and endografts were associated with a significantly higher 30-day mortality (hazard ratio, 8.1; *P* = .016 and hazard ratio, 5.8; *P* = .035, respectively).
- **Take Home Message:** Infected aortic endografts have lower short and long-term survival as well as a lower rate of long-term intervention-free survival when compared with surgical graft and primary aortic infections.
