# Supplementary material for: Aortic endograft infections have worse outcomes compared to aortic surgical grafts or primary mycotic aortic infections
Source: J Vasc Surg. Author manuscript; Available in PMC 2026 Apr 9. (PMC13065352; doi:10.1016/j.jvs.2025.06.011)
Supplement: Supp Figure 1 [file NIHMS2123854-supplement-Supp_Figure_1.pdf]

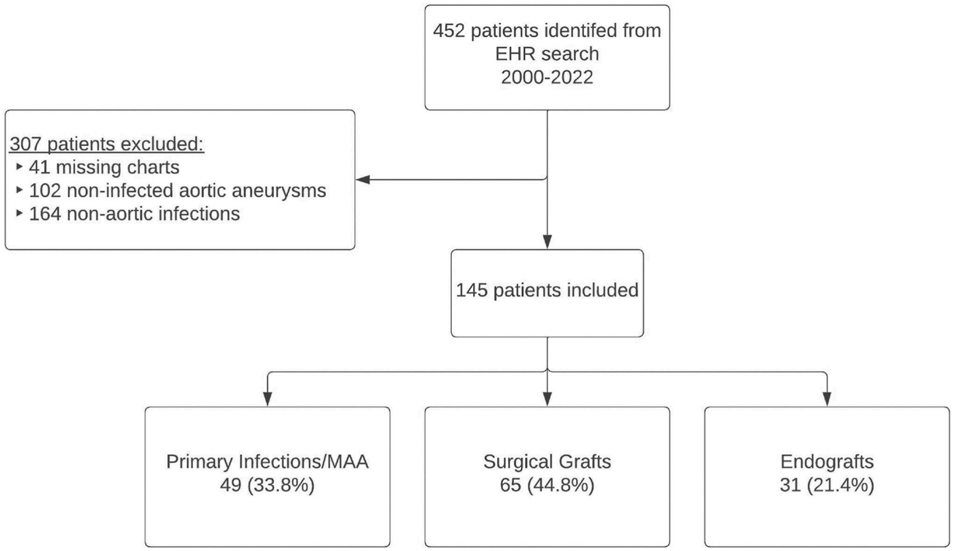

Supplementary Fig 1 (online only). Consort diagram. *EHR*, Electronic health records; *MAA*, mycotic aortic aneurysm.
