# Supplementary material for: Aortic endograft infections have worse outcomes compared to aortic surgical grafts or primary mycotic aortic infections
Source: J Vasc Surg. Author manuscript; Available in PMC 2026 Apr 9. (PMC13065352; doi:10.1016/j.jvs.2025.06.011)

Supplementary Fig 2 (online only). Reintervention-free survival at 1 year (A) and 5 years (B).

Hide Caption
Download
See figure in Article

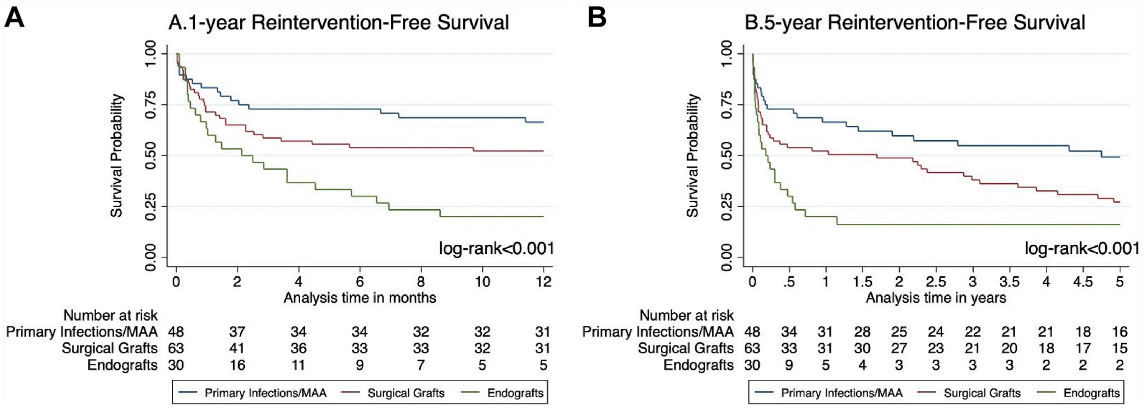

Supplement: Supp Figure 2 [file NIHMS2123854-supplement-Supp_Figure_2.pdf]
