# Supplementary material for: Aortic endograft infections have worse outcomes compared to aortic surgical grafts or primary mycotic aortic infections
Source: J Vasc Surg. Author manuscript; Available in PMC 2026 Apr 9. (PMC13065352; doi:10.1016/j.jvs.2025.06.011)
Supplement: Supp Table I [file NIHMS2123854-supplement-Supp_Table_I.pdf]

Supplementary Table I (online only) Treatment data and infection characteristics

	Primary infections/MAAs n = 49 (33.8%)	Surgical grafts n = 65 (44.8%)	Endografts n = 31 (21.4%)	*P* value
Treatment				
OAR	17 (34.7)	13 (20)	8 (25.8)	.002
EAR	15 (30.6)	31 (47.7)	16 (51.6)	
EVAR	16 (32.7)	8 (12.3)	1 (3.2)	
Medical treatment	0 (0)	5 (7.7)	3 (9.7)	
Palliative treatment	0 (0)	2 (3.1)	1 (3.2)	
Other treatments	1 (2)	6 (9.2)	2 (6.5)	
Surgical culture results				
Fungal	0 (0)	2 (5)	1 (4)	.015
Gram-negative	4 (13)	9 (21)	1 (4)	
Gram-positive	10 (32)	7 (17)	9 (36)	
Mixed	0 (0)	7 (17)	6 (24)	
No growth	17 (55)	17 (40)	8 (32)	
Blood culture results				
Fungal	0 (0)	1 (2)	0 (0)	.014
Gram-negative	4 (9)	2 (4)	2 (7)	
Gram-positive	15 (35)	5 (9)	6 (21)	
No growth	24 (56)	47 (85)	20 (71)	
Antibiotics given	20 (41)	64 (98)	31 (100)	<.001
Antibiotic treatment duration, days	42 (9-96)	45 (30-61)	44 (29-74)	.68
Intraoperative purulence noted	23 (69.7)	27 (55)	25 (96)	<.001
Concurrent procedures done	16 (33)	29 (50)	14 (52)	.12
Timing of revascularization	0 (0-0)	0 (−1 to 0)	0 (0-0)	.002
Operative time, minutes	270 (188-420)	355 (238.5-490)	366 (280-480)	.080
Estimated blood loss, mL	900 (400-2400)	1000 (300-2300)	1450 (700-2900)	.30
Transfusion	39 (83)	37 (80)	26 (96)	.16
Total length of stay, days	14 (11-21)	17 (12-28)	19 (12-31)	.17

	**Primary infections/MAAs n = 49 (33.8%)**	**Surgical grafts n = 65 (44.8%)**	**Endografts n = 31 (21.4%)**	***P* value**
Discharge disposition				
Home	25 (51)	23 (35)	7 (23)	.009
Skilled nursing facility/rehab	19 (39)	30 (46)	10 (32)	
Dead	5 (10)	10 (15)	12 (39)	
Hospice	0 (0)	2 (3)	2 (6)	

***EAR*, Extra-anatomic repair; *EVAR*, endovascular aneurysm repair; *MAA*, mycotic aortic aneurysm; *OAR*, open aneurysm repair in situ.**

Data are presented as number (%) or median (interquartile range).
