# Supplementary material for: Aortic endograft infections have worse outcomes compared to aortic surgical grafts or primary mycotic aortic infections
Source: J Vasc Surg. Author manuscript; Available in PMC 2026 Apr 9. (PMC13065352; doi:10.1016/j.jvs.2025.06.011)
Supplement: Supp Table II [file NIHMS2123854-supplement-Supp_Table_II.pdf]

Supplementary Table II (online only) Multivariable Cox Regression for 30-day all-cause mortality and disease-related mortality

	30-day all-cause mortality			30-day disease-related mortality		
	aHR	95% CI	*P* value	aHR	95% CI	*P* value
Primary infections/MAAs	Ref.	Ref.	Ref.	Ref.	Ref.	Ref.
Surgical grafts	8.1	1.47-44.03	.016	4.9	0.85-28.55	.075
Endografts	5.8	1.13-29.32	.035	5.7	1.09-29.18	.038
Visceral involvement	2.6	0.69-9.94	.152	4.3	1.05-17.61	.042
Treatment						
OAR	Ref.	Ref.	Ref.	Ref.	Ref.	Ref.
EAR	1.4	0.47-4.24	.539	1.4	0.39-4.67	.629
EVAR	3.29	0.31-39.98	.329	2.5	0.22-28.74	.45
Surgical culture results						
No growth	Ref.	Ref.	Ref.	Ref.	Ref.	Ref.
Gram-negative	0.8	0.16-3.66	.75	0.7	0.11-4.96	.721
Gram-positive	2.9	0.76-10.85	.118	4.2	0.96-18.57	.057
Mixed	1.1	0.25-4.72	.919	0.9	0.13-5.58	.879

	30-day all-cause mortality			30-day disease-related mortality		
	aHR	95% CI	*P* value	aHR	95% CI	*P* value
Blood culture results						
No growth	Ref.	Ref.	Ref.	Ref.	Ref.	Ref.
Gram-negative	1.4	0.14-13.37	.788	2.3	0.19-26.67	.506
Gram-positive	2.2	0.46-10.78	.315	1.8	0.35-8.69	.491

***aHR*, Adjusted hazard ratio; *CI*, confidence interval; *EAR*, extra-anatomic repair; *EVAR*, endovascular aneurysm repair; *MAA*, mycotic aortic aneurysm; *OAR*, open aneurysm repair in situ; *Ref*, reference group.**
