# Supplementary material for: Aortic endograft infections have worse outcomes compared to aortic surgical grafts or primary mycotic aortic infections
Source: J Vasc Surg. Author manuscript; Available in PMC 2026 Apr 9. (PMC13065352; doi:10.1016/j.jvs.2025.06.011)
Supplement: Supp Table III [file NIHMS2123854-supplement-Supp_Table_III.pdf]

Supplemental Table III (online only) Baseline demographics of infected grafts subgroups by treatment modality

	OAR<br>21 (21.9%)	EAR<br>47 (48.9%)	EVAR<br>9 (9.4%)	Other<br>19 (19.8%)	*P* value
Age, years	64.6 (12.6)	70.2 (9.2)	68.4 (11.1)	73.6 (12.1)	.067
Gender					
Male	18 (86)	29 (62)	8 (89)	14 (74)	.14
Female	3 (14)	18 (38)	1 (11)	5 (26)	
Race					
Non-Hispanic White	20 (95)	42 (89)	8 (89)	16 (84)	.63
Black	0 (0)	1 (2)	0 (0)	2 (11)	
Other	1 (5)	4 (9)	1 (11)	1 (5)	
Peripheral artery disease	7 (33)	25 (53)	2 (22)	6 (32)	.16
Hypertension	16 (76)	30 (64)	7 (78)	15 (79)	.58
Diabetes	1 (5)	5 (11)	2 (22)	4 (21)	.29
Chronic kidney disease	2 (10)	5 (11)	1 (11)	4 (21)	.66
Hyperlipidemia	11 (52)	22 (47)	1 (11)	7 (37)	.16
Coronary artery disease	6 (29)	18 (38)	3 (33)	8 (42)	.85

	OAR 21 (21.9%)	EAR 47 (48.9%)	EVAR 9 (9.4%)	Other 19 (19.8%)	*P* value
Congestive heart failure	1 (5)	4 (9)	2 (22)	0 (0)	.18
COPD	4 (19)	12 (26)	3 (33)	6 (32)	.78
Recent infection (within 3 weeks)	6 (29)	10 (21)	1 (11)	4 (21)	.82
Recent surgery (within 3 weeks)	2 (10)	7 (15)	1 (11)	1 (5)	.84
Antibiotics on presentation	5 (24)	16 (34)	3 (33)	3 (16)	.48
Asymptomatic presentation	1 (5)	4 (9)	1 (11)	1 (5)	.94
Abdominal pain	7 (33)	19 (40)	4 (44)	3 (16)	.24
Back pain	4 (19)	9 (19)	1 (11)	2 (11)	.87
Melena/GI bleed	2 (10)	4 (9)	5 (56)	3 (16)	.007
Groin symptoms	3 (14)	10 (21)	1 (11)	3 (16)	.89
Constitutional symptoms	5 (24)	12 (26)	2 (22)	4 (21)	1.00
Fever	7 (33)	7 (15)	0 (0)	4 (21)	.15
Heart rate on presentation, bpm	83 (77-88)	85 (73-96)	91 (73-95)	84 (75-92)	.78
SBP on presentation, mmHg	131 (121-161)	131 (118-152)	128 (110-140)	113 (110-135)	.21
Temperature on presentation	36.7 (36.5-37.1)	36.8 (36.5-37.1)	35.7 (35.1-36.8)	36.7 (36.5-37.4)	.10

	OAR<br>21 (21.9%)	EAR<br>47 (48.9%)	EVAR<br>9 (9.4%)	Other<br>19 (19.8%)	*P* value
Rupture on CT	1 (6)	2 (5)	3 (38)	1 (6)	.040
Pseudoaneurysm on CT	1 (6)	7 (19)	3 (43)	3 (18)	.17
Aneurysm size, mm	58.5 (41-86)	44.5 (34.5-55)	37 (32-65)	59 (53-61)	.31
Aneurysm shape					
Saccular	0 (0)	5 (50)	2 (50)	1 (25)	.42
Fusiform	4 (100)	5 (50)	2 (50)	3 (75)	
Aneurysm location					
Suprarenal	3 (15)	2 (4)	2 (22)	0 (0)	.011
Infrarenal	10 (50)	39 (83)	6 (67)	12 (63)	
Pararenal	3 (15)	5 (11)	1 (11)	2 (11)	
Thoracic	4 (20)	1 (2)	0 (0)	5 (26)	

***CT,* Computed tomography; *COPD,* chronic obstructive pulmonary disease; *EAR,* extra-anatomic repair; *EVAR,* endovascular aneurysm repair; *GI,* gastrointestinal; *MAA,* mycotic aortic aneurysm; *OAR,* open aneurysm repair in situ; *SBP,* systolic blood pressure.**

Data are presented as number (%), mean (standard deviation), or median (interquartile range).
